# Rehabilitation Programs for Bedridden Patients with Prolonged Immobility: A Scoping Review Protocol

**DOI:** 10.3390/ijerph182212033

**Published:** 2021-11-16

**Authors:** Vitor Parola, Hugo Neves, Filipa Margarida Duque, Rafael A. Bernardes, Remy Cardoso, Carla A. Mendes, Liliana B. Sousa, Paulo Santos-Costa, Cândida Malça, Rúben Durães, Pedro Parreira, João Apóstolo, Arménio Cruz

**Affiliations:** 1 The Health Sciences Research Unit: Nursing (UICISA: E), Nursing School of Coimbra (ESEnfC), 3000 Coimbra, Portugal; hugoneves@esenfc.pt (H.N.); margaridaduquee@esenfc.pt (F.M.D.); rafaelalvesbernardes@esenfc.pt (R.A.B.); remycardoso@esenfc.pt (R.C.); calexandracmendes@gmail.com (C.A.M.); baptliliana@esenfc.pt (L.B.S.); paulocosta@esenfc.pt (P.S.-C.); parreira@esenfc.pt (P.P.); apostolo@esenfc.pt (J.A.); acruz@esenfc.pt (A.C.); 2 Portugal Centre for Evidence Based Practice: A JBI Centre of Excellence (PCEBP/JBI), 3000 Coimbra, Portugal; 3 Mechanical Engineering Department, Institute of Engineering (ISEC), Polytechnic Institute of Coimbra (IPC), 3030 Coimbra, Portugal; candida@isec.pt; 4 ORTHOS XXI, 4809 Guimarães, Portugal; desenvolvimento.or5@orthosxxi.com

**Keywords:** rehabilitation, rehabilitation exercise, bedridden persons, review

## Abstract

Bedridden patients usually stay in bed for long periods, presenting several motor problems caused by immobility, such as reductions in muscle mass, bone mineral density and physical impairment, resulting in a long recovery process. Thus, identifying physical rehabilitation programs for bedridden patients with prolonged immobility is a matter of urgent research for a solution that will help health professionals and stakeholders to develop more adjusted programs and identify possible gaps. To date, no previous scoping reviews addressing this purpose have been found. This scoping review will be guided by the Joanna Briggs Institute (JBI) methodology, will focus on physical rehabilitation programs for bedridden patients with prolonged immobility and aims to map the programs, the devices used, and the parameters assessed. A relevant set of electronic databases and grey literature will be searched. Data extraction will be conducted using a tool developed by the research team that will address the review objectives and questions. Data synthesis will be presented in tabular form and a narrative summary aligned with the review’s objective. This scoping review will contribute to the improvement of clinical practice, identifying key challenges that might justify the need to develop new programs suitable in clinical and organizational contexts.

## 1. Introduction

Bedridden patients are usually kept in bed for long periods, presenting numerous motor problems caused by immobility, particularly evidencing rapid reductions in muscle mass, bone mineral density and physical impairment [1,2]. Other problems may arise, such as pressure sores, muscular weakness/atrophy, muscular shortness, respiratory complications, blood circulation difficulties and bone demineralization, which can quickly develop and result in long recovery processes. Muscle weakness is particularly eminent in skeletal muscle depletion after days of admission [3]. The skeletal system is also influenced, as the bone metabolism is greatly affected in the absence of proper stimulation [4], or even the cardiovascular system, with atrophy of the baroreceptor function [5].

One of the main complications, particularly frequent among older adults and characterized by muscle mass and function loss, is sarcopenia [6,7]. Its prevalence among this population increases in rehabilitation units up to 34% and 50% [6,8,9], thus denoting the need to develop and explore new interventions, particularly for bedridden patients, because, due to reduced immobility, they are potentially more exposed to this situation. In fact, immobilization directly and negatively affects the quality-of-life outcomes during and after admission [10], and has become a relevant aspect for the prevention of any related complications.

As early mobilization is an important intervention among these patients, programs focusing on specific populations can have positive effects [11]; nevertheless, suitable, structured and efficient programs seem to be lacking.

Although having a hypothetical efficiency, according to Fossat et al. [12], early in-bed interventions, even using leg cycling exercises and electrical muscle stimulation (EMS) of the quadriceps, for example, seem to have no influence on the global muscle strength at discharge from the ICU. On the other hand, McGlinchey et al. [13] concluded that early rehabilitation interventions seem to be no more effective than the usual care. 

Furthermore, Arias-Fernández et al. [14] found that early rehabilitation was associated with significant increases of patients’ functional capacity and muscle strength, with improved outcomes in walking distances and quality-of-life after discharge. In fact, it has been reported in the literature that cycle ergometry and EMS can be used to, at least, maintain muscle strength [15].

This disparity of rehabilitation outcomes may result from different interventions being used without personalization toward the bedridden patient. Several programs have been implemented to address these issues but with no specificity or clinical parameter definition and adequate monitoring. For example, Elsawy et al. [16] implemented a program with at least 10 min of moderate-intense physical activity, starting with walking-like activities. Pithon-Curi et al. [17] proposed an aerobic program, including muscle strength, flexibility and balance exercises, to maximize the program outcomes. Bruseghin et al. [18] adverted that the exercise intensity should be moderate, with a maximum cardiac frequency of 55–70% of the base values, while Lee et al. [19] suggested resistance training combined with a mean exercise plan of moderate intensity, with 8–10 activities for the main muscle groups.

Regarding the clinical parameters and respective biofeedback vigilance, the heart rate variability (HRV) seems to be essential during monitoring [20,21,22], although there are no reference values [23]. The maximum oxygen intake (VO_2_ max) and maximum respiratory rate are also important to define exercise programs but have no determined values to be assessed.

According to Parry et al. [2], there is a growing interest in using assistive technologies, like cycle ergometry and EMS, to empower rehabilitation plans, namely in bedridden patients. These new technologies have been showing important results in the recovery of motor functions [24].

In this regard, some studies have been carried out on physical rehabilitation programs for bedridden patients [25,26,27]. For example, in these studies, bedridden patients were subjected to physical rehabilitation programs once a day for 50 min, five times a week, including hand micro-vibration therapy [25]. However, this program was different from those used in other studies, with the implementation, for example, of EMS [27] or, with movement in six joints, performed five times per joint twice per day and six days per week for four weeks [26]. Additionally, the parameters evaluated are distinct in the available evidence, including the range of motion (ROM) of the hip joint, knee joint and ankle joint [25]; lumbar spine and hip bone mineral density and whole-body lean tissue mass; urine and blood markers of bone metabolism [28]; brain activity [29]; venous flow volume and velocity [30]; joint angle [26] or muscle strength [27]. Consequently, information on physical rehabilitation programs, their characteristics, contexts of application and populations are dispersed in the literature [25,26,27], which hinders the formulation of accurate questions on the effectiveness of those programs and, therefore, the conduct of a systematic review. Moreover, it is recognized that distinct programs have been implemented in different contexts [26,27]; yet, a summary of the physical rehabilitation programs implemented for bedridden patients does not exist.

Thus, the purpose of this mapping will be to clarify the above characteristics. Without this clarification, it is not feasible to advance to a systematic review on their effectiveness. Consequently, relevant issues regarding the nature of the evidence in this area must be addressed before formulating an accurate question on effectiveness. This scoping review aims to respond to these issues.

This scoping review will be guided by the methodology proposed for Joanna Briggs Institute (JBI) to conduct scoping reviews and aims to map the literature related to the physical rehabilitation programs for bedridden patients with prolonged immobility. The main objective of scoping reviews is to explore the literature’s breadth and depth, map and summarise the evidence, help inform future research, identify and address knowledge gaps [31]. However, information on these programs, their characteristics, parameters assessed, contexts of application and devices used is dispersed in the literature. This scoping review will allow us to assess and understand the extent of the knowledge in this field and identify, map, report and hopefully discuss the characteristics of this concept.

An initial search of MEDLINE (via PubMed), the Cochrane Database of Systematic Reviews, the JBI Evidence Synthesis, PROSPERO, JBI EBP Database (via Ovid) and Open Science Framework (OSF) showed that, presently, there is no scoping or systematic review (published or in progress) on this topic [32,33,34].

Mapping the physical rehabilitation programs for bedridden patients with prolonged immobility can significantly contribute to understanding this phenomenon, helping health professionals and stakeholders develop more adjustable programs and which parameters can be used. Therefore, this map will identify relevant issues to help advance evidence-based rehabilitation interventions, develop knowledge, identify possible gaps, and inform systematic reviews.

### Review Question(s)

The main objective of this scoping review is to establish physical rehabilitation programs for bedridden patients with prolonged immobility. More specifically, this scoping review sought to answer the following questions:

1. What are the physical rehabilitation programs for bedridden patients (e.g., neurological, orthopaedic, and cardiorespiratory) with prolonged immobility?
2. What are the rehabilitation domains of the physical rehabilitation programs (motor, respiratory and cardiorespiratory)?
3. What are the parameters assessed during the implementation of the physical rehabilitation programs (e.g., muscle mass and oxygen saturation)?
4. What is the context where the physical rehabilitation programs are implemented (e.g., institutions, community care and outpatient)?
5. What kind of devices are used for bedridden patients with prolonged immobility (e.g., elastics, weights, crankset, and EMS)?

## 2. Methods and Analysis

Scoping reviews are a progressively common methodology for decision-making and research based on identifying and examining the literature on a given topic. Scoping reviews draw on evidence from every research methodology and include evidence from non-research sources. Scoping reviews provide a comprehensive overview to address broader review questions than conventionally more specific systematic reviews of effectiveness or qualitative evidence [31,34].

The first framework of scoping reviews was published in 2005 by Arksey and O’Malley, followed by a contribution from Levac and colleagues (2010). They reflected upon the proposal of Arskey and O’Malley and provided an update to the framework. However, further detailed, and step-by-step methodological guidance was needed to increase the understanding for authors undertaking a scoping review. In 2013, JBI (and partnership) formed a methodological group to build clear, detailed and comprehensive guidance for conducting scoping reviews [31,34,35].

This scoping review will be developed and guided by the JBI methodology [32,33,34]. Preferred Reporting Items for Systematic Reviews and Meta-Analysis extension for scoping reviews (PRISMA-ScR) guidelines will be also used [36].

Considering the steps of chapter 11 [37], the “*Development of a scoping review protocol*”, we ensured to follow a clear and rigorous review process. As the first stage of a scoping review, the protocol predefines the objectives, methods, and reporting, guaranteeing the transparency of the process. The protocol offers the plan for the scoping review and is important in limiting reporting bias.

Significant amendments made to the protocol will be detailed and published alongside the results of the scoping review.

### 2.1. Inclusion Criteria

The inclusion criteria, defined based on the mnemonic “PCC”, according to the JBI recommendations for scoping reviews, were as follows:

Population: This review will consider studies that include bedridden patients, 18 years or over, with prolonged immobility.

Concept: This review will consider studies that explore physical rehabilitation programs.

Context: This review will consider studies, independently of the country of the study, conducted in any setting.

Types of studies: This scoping review will consider, for inclusion, quantitative, qualitative and mixed method study designs. Additionally, all types of systematic reviews will be considered for inclusion.

### 2.2. Search Strategy

The search strategy will map both published and unpublished primary studies and reviews. Two reviewers will develop the search strategy which will be peer-reviewed by an experienced third one, taking into account the Peer Review of Electronic Search Strategies (PRESS) checklist [38]. The three-step search strategy will be implemented as recommended by the JBI [32,34]_._ An initial limited search was undertaken in MEDLINE (via PubMed) and CINAHL Complete (EBSCOhost) to identify articles on the topic. Consequently, the text words/expressions in the titles and abstracts of relevant articles and the index terms used to describe the articles were used to develop a complete search strategy for MEDLINE (via PubMed) (Table 1). An adjusted search strategy was made to the specificities of each information source. Finally, the reference lists of the articles included in the review will be screened for additional papers.

The studies’ languages will be limited to the ones mastered by the authors: English, Portuguese, and Spanish to guarantee a good quality selection process and data extraction. The databases to be searched will include MEDLINE (via PubMed), CINAHL complete (EBSCOhost), Cochrane Central Register of Controlled Trials (EBSCOhost), Cochrane Database of Systematic Reviews (EBSCOhost), SciELO, Scopus, PEDro, JBI EBP Database (via Ovid), SPORTDiscus with Full Text (EBSCOhost) and Rehabilitation & Sports Medicine Source (EBSCOhost). The search for unpublished studies will include DART-Europe and OpenGrey.

### 2.3. Study Selection

All the records identified through database searching will be retrieved and stored in Mendeley^®^ V1.19.8 (Mendeley Ltd., Elsevier, The Netherlands) and duplicates removed. Afterwards, the citations will be imported into Rayyan QCRI (Qatar Computing Research Institute (Data Analytics), Doha, Qatar) for screening, two independent reviewers will screen the titles and abstracts following a pilot test to determine whether they met the inclusion criteria. Potentially eligible studies will be retrieved in full-text to see whether they potentially meet the inclusion criteria or if the abstract is unclear or the study’s relevance is uncertain. Secondly, the full text of the selected citations will be assessed in detail against the inclusion criteria by two independent reviewers. Full-text studies will be excluded if they did not meet the inclusion criteria. Lastly, the references of all the included studies in the review will be hand searched. Any disagreements between the reviewers will be resolved through discussion or with a third reviewer at each stage of the selection process. If the full-text version of an article is inaccessible, the original authors will be contacted.

The search results will be reported in full in the final scoping review and will be presented in a Preferred Reporting Items for Systematic Reviews and Meta-analyses for Scoping Reviews (PRISMA-ScR) flow diagram [36].

### 2.4. Data Extraction

Extracted data from the included articles will be charted by the two independent reviewers in a framework developed according to the JBI proposed template [32,34] and aligned with the objectives and research questions. A draft extraction tool is provided in Table 2. The draft data extraction tool will be modified as required during data extraction from each included paper. As suggested by Levac, Colquhoun and O’Brien [39], to ensure the consistency of data extraction, a priori pilot charting of the first five to ten studies will be performed by two reviewers independent of each other. If necessary, a third reviewer will resolve any disagreements in data extraction.

In the case of missing data, the study authors will be contacted for additional data information. Since review studies will be included the reviewers will report the primary study in the case of data duplication.

### 2.5. Data Analysis and Presentation

The data collected will be presented in diagrammatic or tabular form, depending on which will be more suitable to the review’s objective. A descriptive summary will be provided regarding the charted results in alignment with this scoping review’s objective [32,34].

## 3. Discussion

This scoping review will focus on the physical rehabilitation programs’ contents, feasibility, and potential suitability for bedridden patients with prolonged immobility. A potential strength of this scoping review will be the possibility to include a diverse body of literature, conveying a significant number and variety of patients with such clinical singular conditions that otherwise would not be accessible. We hope to identify research gaps and bring together literature from different disciplines, including intervention programmes, with emerging evidence.

A potential limitation of this scoping review will be that only studies published in English, Portuguese, and Spanish will be included. Articles published in other languages could also be important to this review. For this purpose, we will not exclude papers based on countries or publication dates to prevent restricting ourselves to program types that were particular to some culture or that could have changed over time. 

Furthermore, since the objective of this scoping review is to map the physical rehabilitation programs for bedridden patients with prolonged immobility, no rating of the methodological quality will be provided, and as a result, practice recommendations will potentially be provided with caution [35]. Although the critical appraisal of the included studies will not be evaluated, since it is not relevant for the scoping review, some limitations will be reported to provide valuable information to future research studies/systematic reviews.

## 4. Conclusions

This scoping review will constitute a valuable basis for the analysis and systematization of the main structure of physical rehabilitation programs for bedridden patients with prolonged immobility. This study will also provide useful information, such as the parameters assessed, the type of devices used, the domains and context. In addition to the input of what to research, this scoping will contribute to the improvement of clinical practice, permitting the identification of key challenges that might justify the need to develop new programs suitable in clinical and organizational contexts, considering the available resources, contributing to more efficient and quality care for patients. In addition, the knowledge generated may support a systematic review of effectiveness or indicate the gaps and areas that need clinical investigation to improve the scientific evidence and patient quality care.

## Figures and Tables

**Table 1:** Search strategy for MEDLINE (PubMed). The search was conducted on 7 October 2021.

Search	Query	Record Retrieved
#1	((((rehabilitation[MeSH Terms]) OR (rehabilitation[Title/Abstract])) OR (((((exercise*[Title/Abstract]) OR (Exercise Movement Techniques[MeSH Terms])) OR (Rehabilitation Exercise[MeSH Terms])) OR (Exercise[MeSH Terms]))	820,263
#2	((bedridden[Title/Abstract]) OR (bedridden persons[MeSH Terms]))	2773
#3	(((bedridden[Title/Abstract]) OR (bedridden persons[MeSH Terms]))) AND (“rehabilitation”[MeSH Terms] OR “rehabilitation”[Title/Abstract] OR “exercise*”[Title/Abstract] OR “exercise movement techniques”[MeSH Terms] OR “exercise therapy”[MeSH Terms] OR “exercise”[MeSH Terms])	434
#4	(((bedridden[Title/Abstract]) OR (bedridden persons[MeSH Terms]))) AND (“rehabilitation”[MeSH Terms] OR “rehabilitation”[Title/Abstract] OR “exercise*”[Title/Abstract] OR “exercise movement techniques”[MeSH Terms] OR “exercise therapy”[MeSH Terms] OR “exercise”[MeSH Terms]) Filters: English, Portuguese, Spanish, MEDLINE	266

**Table 2:** Data extraction tool.

Scoping Review Details	
Scoping review title	Physical rehabilitation programs for bedridden patients with prolonged immobility: A Scoping Review
Review objective(s)	To map the literature related to the physical rehabilitation programs for bedridden patients with prolonged immobility.
Review question(s)	What are the physical rehabilitation programs for bedridden patients (e.g., neurological, orthopaedic, and cardiorespiratory) with prolonged immobility? What are the rehabilitation domains of the physical rehabilitation programs (motor, respiratory and cardiorespiratory)? What are the parameters assessed during the implementation of the physical rehabilitation programs (e.g., muscle mass and oxygen saturation)? What is the context where the physical rehabilitation programs are implemented (e.g., institutions, community care and outpatient)? What devices are used for bedridden patients with prolonged immobility (e.g., elastics, weights, crankset, and EMS)?
**Inclusion/exclusion criteria**	
Population	This review will consider studies that include bedridden patients,18 years or over, with prolonged immobility.
Concept	This review will consider studies that explore the physical rehabilitation programs.
Context	This review will consider studies independently of the country of the study conducted in any setting.
Types of evidence source	This scoping review will consider, for inclusion, the quantitative, qualitative and mixed method study designs. Additionally, all types of systematic reviews will be considered for inclusion.
**Evidence source details and characteristics**	
Author(s)	
Year of publication	
Origin/country of origin (where the source was published or conducted)	
Aims/purpose	
Population and sample size	
**Details/Results extracted from source of evidence** (in relation to the concept of the scoping review)	
Physical rehabilitation programs	
Rehabilitation domains	
Parameters assessed	
Context	
Devices used	
